# Effects of ACEI/ARB in hypertensive patients with type 2 diabetes mellitus: a meta-analysis of randomized controlled studies

**DOI:** 10.1186/1471-2261-14-148

**Published:** 2014-10-25

**Authors:** Guang Hao, Zengwu Wang, Rui Guo, Zuo Chen, Xin Wang, Linfeng Zhang, Wei Li

**Affiliations:** Division of Prevention & Community Health, National Center for Cardiovascular Disease, Fuwai Hospital, Peking Union Medical College & Chinese Academy of Medical Sciences, No. 167 Beilishi Road Xicheng District, Beijing, 100037 China; Chinese medical association publishing house, No.42 Dongsi Street Dongcheng District, Beijing, 100037 China; National Center for Cardiovascular Disease, Fuwai Hospital, Peking Union Medical College & Chinese Academy of Medical Sciences, No. 167 Beilishi Road Xicheng District, Beijing, 100037 China

## Abstract

**Background:**

The effects of angiotensin-converting enzyme (ACE) inhibitors and angiotensin II receptor blockers (ARBs) on cardiovascular (CV) risk in hypertensive patients with type 2 diabetes mellitus (T2 DM) are uncertain. Our objective was to analyze the effects of ACE/ARBs, on the incidence of myocardial infarction, stroke, CV events, and all-cause mortality in hypertensive patients with T2 DM.

**Method:**

PubMed and Embase databases were searched through January 2014 to identify studies meeting a priori inclusion criteria and references in the published articles were also reviewed. Two investigators independently extracted the information with either fixed-effect model or random-effect model to assess the effects of ACE/ARBs treatment in hypertensive patients with T2 DM.

**Results:**

Ten randomized controlled studies were included with a total of 21,871 participants. Overall, treatment with ACE/ARBs in hypertensive patients with T2 DM was associated with a statistically significant 10% reduction in CV events, pooled hazard ratio (HR) of 0.90 [95% confidence intervals (CI): 0.82-0.98] with no heterogeneity (I^2^ = 19.50%; *P* = 0.275);and 17% reduction in CV mortality, pooled HR of 0.83 [95% CI: 0.72-0.96] with no heterogeneity (I^2^ = 0.9%; *P* = 0.388). ACE/ARBs was not associated with MI, stroke and all-cause mortality.

**Conclusions:**

Treatment with ACE/ARBs results in significant reduction in CV events and mortality in hypertensive patients with T2 DM.

**Electronic supplementary material:**

The online version of this article (doi:10.1186/1471-2261-14-148) contains supplementary material, which is available to authorized users.

## Background

Hypertension and type 2 diabetes (T2 DM) frequently coexist, and patients with this combination are at a higher risk for cardiovascular (CV) events than those suffering from hypertension or T2 DM alone [1–3]. Most (60% to 80%) people with T2 DM die of CV complications, and up to 75% of specific CV complications have been attributed to high blood pressure (BP) [4]. The improved treatment of hypertension has been associated with a marked reduction in death and hospitalization from CV disease [5]. The use of angiotensin-converting enzyme (ACE) inhibitors or angiotensin II receptor blockers (ARBs), could reduce both CV morbidity and mortality across populations that apart from hypertension [6–8], had other co-morbid conditions.

The beneficial effect of ACE inhibitor treatment on all-cause mortality for hypertensive patients was well established in a recent meta-analysis [9]. However, the effect of ACE/ARBs on CV risk in hypertensive patients with T2 DM remains controversial. The Heart Outcomes Prevention Evaluation (HOPE) study showed that treatment with Ramipril reduced cardiovascular events in patients with diabetes, out of which 56% were hypertensive [10]. The Fosinopril Versus Amlodipine Cardiovascular Events Randomized Trial (FACET) and Captopril Prevention Project (CAPPP) study demonstrated that the ACE inhibitors fosinopril could significantly reduce risk of major vascular events in hypertensive diabetic patients compared with controls [11, 12]. However, other studies like the Irbesartan Diabetic Nephropathy Trial (IDNT) or The Action in Diabetes and Vascular disease: preterAx and diamicroN-MR Controlled Evaluation (ADVANCE) trial failed to find such a beneficial effect in hypertensive patients with T2 DM [13, 14].

To our best knowledge, there is no meta-analysis or RCT focused on the effect of ACE/ARBs on CV risk in hypertensive patients with T2 DM, although these classes of drug were recommended for these patients by the guidelines of 2013 European Society of Hypertension (ESH) and of the European Society of Cardiology (ESC) and the eighth report of Joint National Committee (JNC 8) [15, 16]. However, the evidence derived from papers focused on the Individuals with and without Diabetes Mellitus separately [6].

The objective of the present study is to review randomized clinical trials (RCT) were revising the effect of antihypertensive treatment using ACE/ARBs on incidence of myocardial infarction (MI), stroke, CV events, and all-cause mortality in hypertensive patients with T2 DM.

## Methods

### Search strategy and study selection

**Figure 1:**
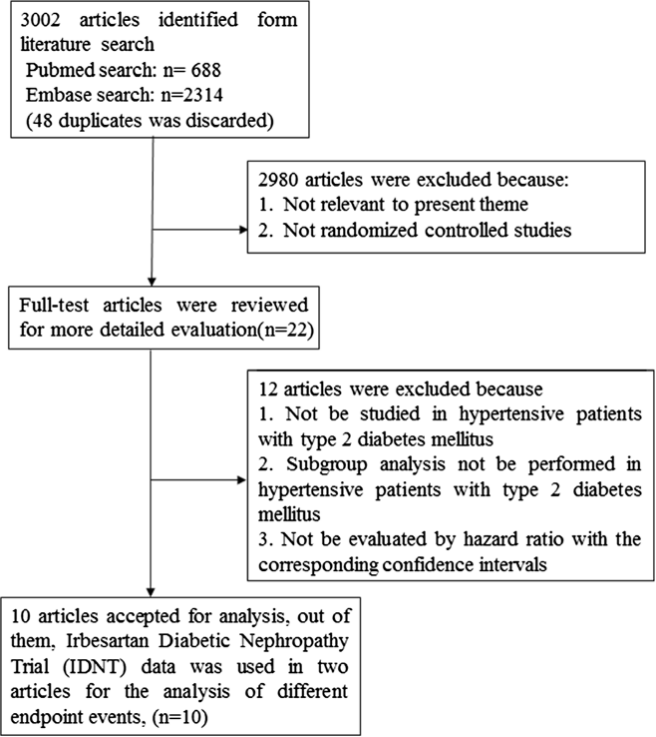
**Flow chart of study selection.**

We performed a systematic search of Pubmed and Embase databases through January 2014 for relevant studies performed in hypertensive patients with T2 DM. Subject headings and key words used for the literature search were as follows: 1) mortality, CV diseases, MI and stroke; 2) hypertension and diabetes; 3) angiotensin-converting enzyme inhibitors and angiotensin receptor blockers; 4) RCTs. The titles, abstracts and full-texts were reviewed independently by two reviewers. The criteria for eligible studies were as follows: 1) Randomized clinical trials in hypertensive patients with T2 DM comparing active treatment with ACE inhibitors or ARBs with control treatment (placebo, life style changes, active antihypertensive treatment with drugs other than ACEI or ARB); 2) The endpoints were mortality, CV events, MI or stroke; 3) Hazard ratios (HR) were calculated with the corresponding confidence intervals (CI). Following this search, references of published articles were also reviewed. Finally, 10 RCTs were selected, out of them, IDNT data was used in two articles for the analysis of different endpoint events [14, 17] (Figure 1).

### Data extraction

We collected the following information from each study: first author name or study title, year of publication, country of origin, gender, follow-up period, class of anti-hypertensive drugs, disease outcome, the number of trial participants, HR with the corresponding 95% CI, and the mean diastolic and systolic BP at baseline. Two investigators independently calculated and tabulated the data using a standard extraction formula. Any discrepancies were discussed by the research team and were resolved. In addition, we used the modified Jadad scale to determine the quality of the studies [18].

### Endpoint definition

The endpoints of this pooled analysis were MI, stroke, CV events, and CV and all-cause mortality. Stroke including fatal or nonfatal stroke, MI including fatal or nonfatal acute MI, and CV events is according to trial-specific definitions, including coronary artery bypass, percutaneous transluminal coronary angioplasty, death from cardiovascular causes, nonfatal myocardial infarction, heart failure, etc. Not all studies included every endpoint. Data on all-cause mortality were available in 7 trials, CV mortality in4 trials, CV events in 8 trials, MI in 5 trials, and stroke in7 trials.

### Statistical analysis

The combined risk estimates were computed with either fixed-effect model or random-effect model, if heterogeneity existed. The Cochrane Q statistics (significance level of *P* < 0.10) and the I^2^ statistics were used to assess the heterogeneity of HR across studies. Since the characteristics were not consistent from study-to-study, we further conducted sensitivity analysis and stratified analyses to explore the possible explanation for heterogeneity. For sensitivity analysis, the effect of a single study on overall risk factors was investigated by omitting one study at a time. Stratified analyses were performed by control group (placebo-controlled or active antihypertensive drugs, baseline systolic BP, reduction of systolic BP, and class of antihypertensive drugs (ACE inhibitors or ARBs). The possibility of publication bias was assessed using the Begg and Egger test. We also performed the visual inspection of Begg funnel plots in which log RRs were plotted against their SEs. All analyses were conducted using STATA version 11.2 (Stata Corp LP, College Station, TX). A value of *P* < 0.05 was defined as statistically significant.

## Results

### Characteristics of the studies

Table 1 shows the 10RCTs’ characteristics of 21,871 hypertensive patients with T2 DM, published between 1998 and 2012. Two RCTs were conducted in the United States [11, 17], 3 in Europe [12, 19, 20], 2 in Japan [21, 22], and 3 were multicenter studies [13, 14, 23]. IDNT data was used in two articles for the analysis of different endpoint events [14, 17]. The length of follow-up ranged from 2.5 to 9 years. All studies were conducted in hypertensive patients with T2 DM except for one (the respondents were diabetics, 59% of those were hypertensive) [13]. The number of respondents ranged from 257 to 11,140 (total 21,871) participants. BP decreased more in ACE/ARBs group (mean difference: systolic BP 4.14 mm Hg and diastolic BP 1.63 mmHg) compared with those assigned to control treatment. Study quality generally was good (Additional file 1: Table S1).

**Table 1:** **Baseline characteristics of study population in 10 trials**

Study/author	Public year	Country	Age (year)	Total (I/C)*	Female (%)	follow-up years	Medications used (I/C)*	BP (mmHg) I(SBP/DBP; C(SBP/DBP	Reduction of BP (mmHg) ^#^I(SBP/DBP; C(SBP/DBP)
UKPDS [19]	1998	United Kingdom	56	400/358	48	over 9	Captopril/Atenolol	159/94; 159/93	15/10; 16/12
FACET [11]	1998	United State	63	189/191	41	2.5-3.5	Fosinopril/Amlodipine	170/95; 171/94	19/8; 13/8
RENAAL [23]	2001	Multi-centers	60	751/762	37	3.4	Losartan/Placebo	152/82; 153/82	12/8; 11/8
CAPPP [12]	2001	Sweden/Finland	25–66	309/263	38	6.1	Captopril/Conventional drugs	164/97; 163/97	8/4; 10/5
IDNT [14, 17]&	2001/2003	Multi-centers	30-70	579/569	32	2.6	Irbesartan/Placebo	160/87; 158/87	20/10; 16/7
ADVANCE [13]	2007	Multi-centers	66	5,569/5,571	43	4.3	Perindopril/Placebo	145/81; 145/81	5.6/2.2^#^
CASE-J [21]	2010	Japan	64	1,011/1,077	44	3.3	Candesartan/Amlodipine	160/88; 160/88	---
DEMAND [20]	2011	Italy/Slovenia	≥40	127/127	37	3.8	Delapril/Placebo	147/87; 147/88	11/6; 9/5
NHS [22]	2012	Japan	63	575/575	34	3.2	Valsartan/Amlodipine	145/82; 144/81	14/9; 13/8

UKPDS = United Kingdom Prospective Diabetes Study Group, FACET = Fosinopril Versus Amlodipine Cardiovascular Events Randomized Trial, RENAAL = Reduction of Endpoints in NIDDM with the Angiotensin II Antagonist Losartan, CAPPP = The Captopril Prevention Project, IDNT = Irbesartan Diabetic Nephropathy Trial, ADVANCE = The Action in Diabetes and Vascular disease: preterAx and diamicroN-MR Controlled Evaluation, CASE-J = candesartan antihypertensive survival evaluation in Japan, DEMAND = Delapril and Manidipine for Nephroprotection in Diabetes, NHS = NAGOYA HEART Study.

*I/C = Intervention/Control, SBP = systolic blood pressure, DBP = diastolic blood pressure; ^#^reduction of SBP/DBP in intervention group compared with in placebo; ----- not give detail information; &IDNT data was used in two articles for the analysis of different endpoint events.

### All-cause mortality

**Figure 2:**
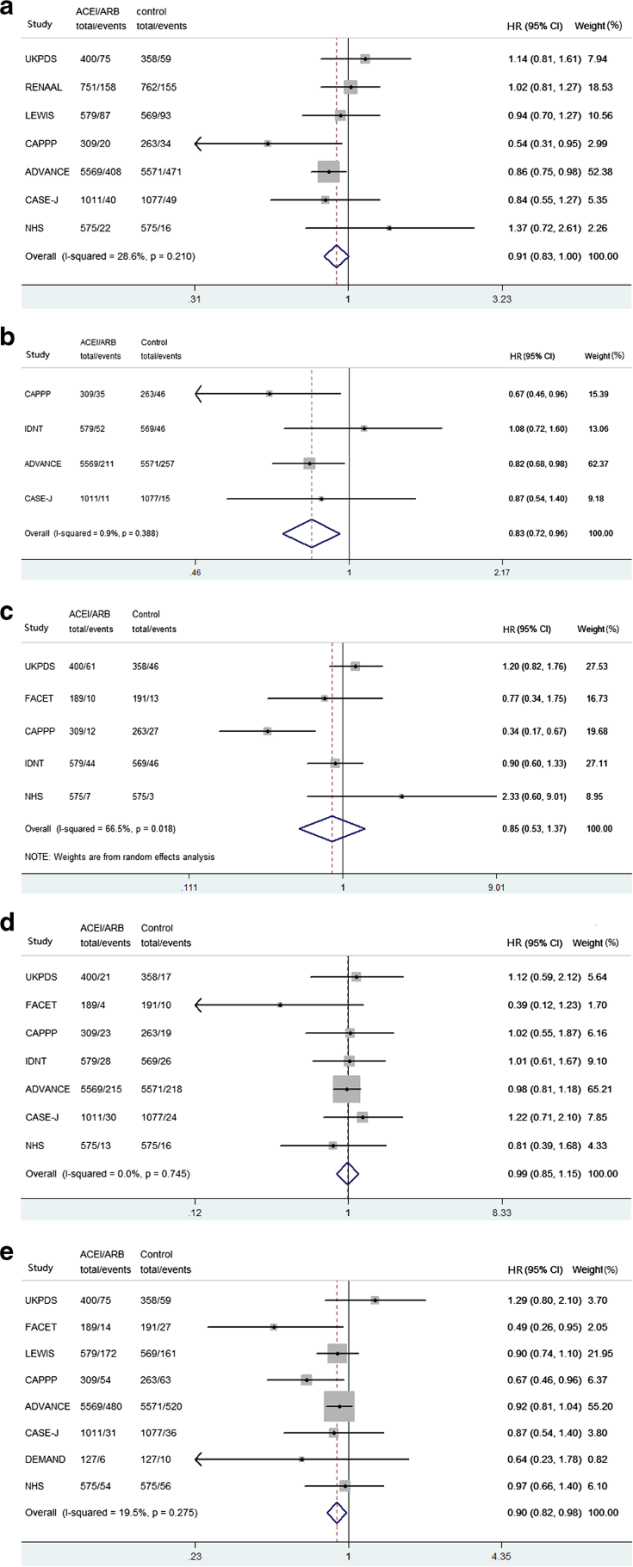
**All-cause mortality (a), CV mortality (b), MI (c), stroke (d), and CV events (e) treatment effect of ACE/ARBs in all included trials.** CV = cardiovascular, MI = myocardial infarction, HR = hazard ratio, CI = confidence interval.

Treatment with ACE/ARBs did not reduce significantly all-cause mortality (HR: 0.91, 95% CI: 0.83-1.00, *P* = 0.062); the degree of heterogeneity in the treatment effect across all trials was low (I^2^ = 21.0%) and non-significant (*P* = 0.210, Figure 2a).

### CV mortality

Treatment with RAAS achieved a 17% significant reduction in CV mortality (HR: 0.83, 95% CI: 0.72-0.96, *P* = 0.012), the degree of heterogeneity in the treatment effect across all trials was low (I^2^ = 0.9%) and non-significant (*P* = 0.388, Figure 2b).

### MI and stroke

In all 5 trials grouped together, MI risk was not reduced significantly (HR: 0.85, 95% CI: 0.53-1.37, *P* = 0.511), with a higher heterogeneity across all trials (I^2^ = 66.5%, *P* = 0.018, Figure 2c). In all 8 trials grouped together, we did not find a statistically significant reduction in stroke with ACE/ARBs treatment (HR: 0.99, 95% CI: 0.85-1.15, *P* = 0.855); the degree of heterogeneity in the treatment effect across all trials was low (I^2^ = 0.0%) and non-significant (*P* = 0.745, Figure 2d).

### CV events

In all 8 trials grouped together, treatment with ACE/ARBs was associated with a statistically significant 10% significant reduction in CV events (HR: 0.90, 95% CI: 0.82-0.98, *P* = 0.019), with non-significant heterogeneity between studies (I^2^ = 19.50%, *P* = 0.275, Figure 2).

### Stratified and sensitivity analysis

For stratified analysis of key characteristics see Additional file 1: Table S2. Exclusion of any single study did not substantially alter the result in analysis of different endpoints.

### Publication bias

The funnel-plot asymmetry, and the P-values using an Egger regression test were 0.97 (all-cause mortality), 0.41(CV events), 0.81 (CV mortality), 0.80 (MI) and 0.55 (stroke), indicating no evidence for publication bias.

## Discussion

Although the ESH/ESC and JNC8 [15, 16] recommend ACE inhibitors or ARB as the preferred therapy in hypertensive patients with T2 DM, the effect of ACE/ARBs on mortality in hypertensive patients with T2 DM remains not unequivocally accepted. In an effort to evaluate the effects of ACE/ARBS on CV outcomes in hypertensive patients with T2 DM, we performed a systematic review of the literature and analyzed 10 RCTs. Overall, the results of our review showed that treatment with the ACE/ARBs was associated with a 10% reduction in CV events and, 17% reduction in CV mortality. Previous studies and meta-analyses evaluating the effects of ACE/ARBs on the overall risk for CV events in hypertensive patients have yielded conflicting results. The Asia Pacific Cohort Studies Collaboration (APCSC) study indicated that major CV risk was reduced to a comparable extent in hypertensive individuals with or without diabetes while on an ACE inhibitor-based treatment, or other antihypertensive regimens [24]. The NAVIGATOR Study also failed to find a reduction of CV risk in patients with impaired glucose tolerance and established CV disease using valsartan and lifestyle modification [25]. On the other hand, in another study [26], initiation of antihypertensive treatment involving ACE inhibitors in hypertensive patients and with 8% T2 DM, appeared to lead to better outcomes than treatment with diuretic agents. A recent meta-analysis showed that ACE/ARBs were associated with a 5% reduction in all-cause mortality and a 7% reduction in CV mortality; the treatment effect resulted entirely from the class of ACE inhibitors. No mortality reduction could be demonstrated with ARB treatment in the hypertension population [9]. Bangalore and colleagues pooled 37 RCTs and showed that ARBs reduce the risk of stroke, heart failure, and new onset diabetes compared with controls in general hypertensive [27]. In our study, we could not ascertain whether the observed reduction in CV events (10%) and reduction in CV mortality (17%) among hypertensive patients with T2DM could be was attributed to treatment with ACE inhibitors or treatment with ARBs.

In our meta-analysis, beneficial effects were only found in ADVANCE (for CV and all-cause mortality), FACET (for CV event) and CAPPP (for CV mortality and MI) study, i.e. studies where ACE inhibitors were used [11–13]. However, stratified analysis of all-cause mortality and CV events failed to find an association both with the ACE inhibitor group and the ARB group.

There was marginal statistically significant effect of ACE/ARBs on the all-cause mortality in hypertensive patients with T2DM (*P* = 0.062). A recent meta-analysis also found the ACEIs significantly reduced the risk of all-cause mortality by 13% in diabetes [28]. More studies should be performed to confirm the effect of ACEI/ARBs in hypertensive patients with T2DM.

The beneficial effect on CV events might be attributed mainly to differences in systolic BP [29]. In the Systolic Hypertension in the Elderly Program (SHEP) study, elderly persons with T2 DM derived more benefit from aggressive systolic BP lowering in reduction of CV than those without diabetes [30]. Additional benefit of aggressive BP lowering in the diabetic population was observed in a sub-analysis of Systolic Hypertension in Europe (SystEur) Trial. In that trial, although systolic BP was reduced by a comparable amount in each group, the risk reduction in mortality from CV disease was 13% in non-diabetic patients versus 76% for the diabetic patients [31]. There is weaker evidence that the effect of BP lowering between different drug classes is varied. Law and colleagues [32] pooled 354 RCTs and found that ACE inhibitors, calcium antagonists, and five categories of drug produced similar reductions in BP. We did not find a significant effect of BP lowering in stratified analysis, which may be due to lower statistical power. Therefore, further specific RCTs need to be performed to evaluate the BP lowering effect of Various ACE/ARBs in hypertensive patients with T2DM.

Several limitations of our analysis have to be mentioned. Firstly, our results are subject to limitations inherent to any meta-analysis based on pooling of data from different trials, including use of different definitions for CV events, different dosages of the active and control drug, different follow-up times, optimal BP target, combination treatment and participants with other concomitant conditions or background therapy. Although we could not find a differential result in sensitivity or stratified analyses, it was impossible to accurately estimate the effect of ACE/ARBs in patients with T2 DM. Secondly, one study did not report HR with 95% CI when a significant result was found [33] and it was not included in our analysis. This issue probably resulted in an underestimation of the effect of ACE/ARBs.

## Conclusion

This meta-analysis, which involved almost 21,871 hypertensive patients with T2 DM, demonstrated that ACE/ARBs as a class of antihypertensive drugs were associated with a significant 10% reduction effect of CV events and 17% reduction in CV mortality when compared with control or active antihypertensive regimens with drugs other than ACEI or ARB. Stratified subgroup analysis according to class of BP-lowering regimens and different control group failed to differentiate between ACE inhibitors or ARB effect on CV outcomes. More studies should be performed to clarify if the beneficial effect on CV events and CV mortality among hypertensive patients with T2DM was derived from ACEIs, or ARBs.

## Electronic supplementary material

Additional file 1: Table S1: Risk assessment of bias used modified Jadad score. **Table S2.** Stratified Analyses of Pooled Hazard Ratio of ACEI/ARB and cardiovascular risk. (DOCX 22 KB)
