# Supplementary material for: Effects of ACEI/ARB in hypertensive patients with type 2 diabetes mellitus: a meta-analysis of randomized controlled studies
Source: BMC Cardiovasc Disord. 2014 Oct 25;14:148. doi: 10.1186/1471-2261-14-148 (PMC4221690; doi:10.1186/1471-2261-14-148)
Supplement: Supplementary file 1 — Additional file 1: Table S1: Risk assessment of bias used modified Jadad score. Table S2. Stratified Analyses of Pooled Hazard Ratio of ACEI/ARB and cardiovascular risk. (DOCX 22 KB) [file 12872_2014_793_MOESM1_ESM.docx]

Additional file 1: Table S1 Risk assessment of bias used modified Jadad score

| Study/Author | Randomization | Concealment of  allocation | Double blinding | Withdrawals  and dropouts | scores |
| --- | --- | --- | --- | --- | --- |
| UKPDS | 1 | 1 | 1 | 1 | 4 |
| FACET | 1 | 0 | 1 | 1 | 3 |
| RENAAL | 2 | 1 | 2 | 1 | 6 |
| CAPPP | 2 | 0 | 1 | 1 | 5 |
| IDNT | 1 | 1 | 2 | 1 | 5 |
| ADVANCE | 2 | 1 | 2 | 1 | 6 |
| CASE-J | 2 | 0 | 0 | 1 | 3 |
| DEMAND | 2 | 1 | 1 | 1 | 5 |
| NHS | 2 | 1 | 1 | 1 | 5 |

UKPDS=United Kingdom Prospective Diabetes Study Group, FACET = Fosinopril Versus Amlodipine Cardiovascu-lar Events Randomized Trial, RENAAL = Reduction of Endpoints in NIDDM with the Angiotensin II Antagonist Losartan, CAPPP = The Captopril Prevention Project, IDNT = Irbesartan Diabetic Nephropathy Trial, ADVANCE = The Action in Diabetes and Vascular disease: preterAx and diamicroN-MR Controlled Evaluation, CASE-J = candesartan antihypertensive survival evaluation in Japan, DEMAND = Delapril and Manidipine for Nephroprotection in Diabetes, NHS = NAGOYA HEART Study

Table S2 Stratified Analyses of Pooled Hazard Ratio of RAAS inhibitors and cardiovascular risk.

| Stratified analyses | Pooled HR (95% CI) | Heterogeneity |
| --- | --- | --- |
| All-cause mortality | | |
| Control group  Active treatment  Placebo | 0.92(0.65-1.31)  0.90(0.81-1.00) | I^2^=54.3%; *P*=0.087  I^2^=0.0%; *P* =0.543 |
| Class of hypertensive drugs  ACEI  ARB | 0.91(0.75-1.09)  0.95(0.76-1.20) | I^2^=43.5%; *P* =0.132  I^2^=0.0%; *P* =0.454 |
| Systolic blood pressure  < 160mmHg  ≥ 160mmHg | 0.93(0.83-1.03)  0.83(0.67-1.04) | I^2^=23.3%; *P* =0.266  I^2^=31.9%; *P* =0.230 |
| Reduction of systolic blood pressure  ≤ 12mmHg  > 12mmHg | 0.86(0.71-1.05)  1.05(0.85-1.30) | I^2^=40.7%; *P* =0.167  I^2^=0.0%; *P* =0.495 |
| CV events | | |
| Control group  Active treatment  Placebo | 0.84(0.63-1.12)  0.91(0.83-1.01) | I^2^=55.8%; *P* =0.060  I^2^=0.0%; *P* =0.983 |
| Class of hypertensive drugs  ACEI  ARB | 0.80(0.57-1.13)  0.91(0.81-1.04) | I^2^=67.4%; *P* =0.027  I^2^=0.0%; *P* =0.989 |
| Systolic blood pressure  < 160mmHg  ≥ 160mmHg | 0.94(0.84-1.06)  0.83(0.70-0.97) | I^2^=0.0%; *P* =0.408  I^2^=49.4%; *P* =0.115 |
| Reduction of systolic blood pressure  ≤ 12mmHg  > 12mmHg | 0.85(0.67-1.08)  0.91(0.70-1.20) | I^2^=48.2%; *P* =0.145  I^2^=46.4%; *P* =0.133 |
| Stroke | | |
| Control group  Active treatment  Placebo | 0.99(0.74-1.34)  0.98(0.83-1.17) | I^2^=0.0%; *P* =0.482;  I^2^=0.0%; *P*=0.912 |
| Class of hypertensivedrugs  ACEI  ARB | 0.97(0.82-1.16)  1.04(0.75-1.44) | I^2^=0.0%; *P* =0.046  I^2^=0.0%; *P* =0.672 |
| Systolic blood pressure  < 160mmHg  ≥ 160mmHg | 0.98(0.82-1.17)  1.01(0.74-1.37) | I^2^=0.0%; *P* =0.807  I^2^=1.2%; *P* =0.386 |
| Reduction of systolic blood pressure  ≤ 12mmHg  > 12mmHg | 0.98(0.82-1.18)  0.92(0.66-1.28) | I^2^=0.0%; *P* =0.903  I^2^=0.0%; *P* =0.440 |

RAAS = Renin-angiotensin-aldosterone system, ARB = angiotensin II receptor blocker, ACEI = ngiotensin-converting enzyme inhibitor, CV = cardiovascular, HR = hazard ratio, CI = confidence interval
